# Association of prevalence of electronic cigarette use with smoking cessation and cigarette consumption in England: a time–series analysis between 2006 and 2017

**DOI:** 10.1111/add.14851

**Published:** 2019-12-04

**Authors:** Emma Beard, Robert West, Susan Michie, Jamie Brown

**Affiliations:** ^1^ Department of Behavioural Science and Health University College London London UK; ^2^ Department of Educational, Clinical and Health Psychology University College London London UK

**Keywords:** ARIMAX, e‐cigarettes, Electronic cigarettes, STS, time‐series, tobacco

## Abstract

**Aims:**

To provide up‐to‐date estimates of how changes in the prevalence of electronic cigarette (e‐cigarette) use in England have been associated with changes in smoking cessation activities and daily cigarette consumption among smokers in England.

**Design:**

Time–series analysis of population trends.

**Setting:**

England.

**Participants:**

Participants came from the Smoking Toolkit Study, which involves repeated, cross‐sectional household surveys of individuals aged 16 years and older in England. Data were aggregated on approximately 1200 past‐year smokers each quarter (total *n* = 50 498) between 2007 and 2017.

**Measurements:**

Prevalence of e‐cigarette use in current smokers was used to predict (a) prevalence of quit attempts among last‐year smokers, (b) overall quit rates among last‐year smokers and (c) mean cigarette consumption per day among current smokers. Prevalence of e‐cigarette use during a quit attempt among last‐year smokers was used to predict (a) quit success rate among last‐year smokers and (b) overall quit rates among last‐year smokers.

**Findings:**

Overall quit rates increased by 0.054% [95% confidence interval (CI) = 0.032–0.076, *P* < 0.001] and 0.050% (95% CI = 0.031–0.069, *P* < 0.001) respectively for every 1% increase in the prevalence of e‐cigarette use by smokers and e‐cigarette use during a quit attempt. Quit success rates increased by 0.060% (95% CI = 0.043–0.078, *P* < 0.001) for every 1% increase in the prevalence of e‐cigarette use during a quit attempt. No clear evidence was found for an association between e‐cigarette use and either prevalence of quit attempt (BAdj = 0.011, 95% CI = −0.046 to 0.069, *P* = 0.698) or cigarette consumption (BAdj = 0.019, 95% CI = −0.043 to 0.082, *P* = 0.542).

**Conclusion:**

Changes in prevalence of e‐cigarette use in England have been positively associated with the overall quit rates and quit success rates but not clearly associated with the prevalence of quit attempts and mean cigarette consumption.

## Introduction

The potential for e‐cigarettes to contribute to population health by promoting smoking cessation or reduction remains contested 1. Different types of study design each have strengths and limitations in addressing this issue. Randomized controlled trials (RCTs) may be limited in terms of generalizability, while comparative observational studies are subject to potential bias by unmeasured confounding or selection bias. Population‐level time–series analyses can provide both an important source of triangulating evidence and direct estimates of population impact 2. We report a time–series analysis on the association between the prevalence of e‐cigarette use and smoking cessation and cigarette consumption among remaining smokers in England up to 2017. This follows a prior report of a time–series analysis on quitting activities up to 2015 3.

RCT evidence supports the view that e‐cigarette use in a quit attempt can aid smoking cessation 4, and the effect may be greater than licensed nicotine products 5. One RCT has also found that e‐cigarette use led to greater smoking reduction in those who did not quit than licensed nicotine products 6. Most e‐cigarette use does not involve engagement with health professionals, so the extent to which these results generalize is not known. Evidence from comparative observational studies of e‐cigarette use in quit attempts presents a more mixed picture, with some studies showing higher success rates when e‐cigarettes are used in quit attempts and others failing to find an effect 1, 3, 4, 5, 6, 7. The ability of the study designs to control for unmeasured confounding and selection bias may have played an important role in differences in study findings 8, 9. Other observational studies of past e‐cigarette use, not necessarily in a quit attempt, have found a negative association with subsequent quitting 1; however, this negative association could have arisen from unmeasured confounding or selection bias 7, 10.

Population trend data do not suffer from the limitations of RCTs and comparative observational studies. RCTs are limited by generalizability outside the clinical trial context. Population surveys comparing smokers who use e‐cigarettes with people who use other methods to quit are limited by the possibility that those using e‐cigarettes may be more likely to succeed in quit attempts because of unmeasured confounding variables. Population trend data, by definition, involve the whole population and there can be no individual‐level confounding. Population trend data are limited by the possibility of population‐level confounding, such as introduction of policies that may affect overall quitting rates. In theory, they may also be affected by changes in the demographic or smoking profiles of the population, although it would be implausible for sufficiently large changes to occur to influence the results during time intervals of a year or less used in studies of this kind. These would also not affect clinical trials or comparative observational studies. Thus, the three sources of information together (clinical trials, comparative observational studies and population trend data) provide powerful triangulation on the true effect size of widely used aids to cessation such as e‐cigarettes.

We previously published a time–series analysis on prevalence of e‐cigarette use in England and a range of smoking‐cessation variables up to the first quarter of 2015 3. The current paper extends this time–series analysis for a further eight quarters (2 years) to provide greater clarity on the key outcomes by increasing the power to detect an association between e‐cigarette use and quit attempt, if one exists, allowing greater opportunity to estimate key associations while adjusting for potential confounders 11. Recent years have also seen a slowing down in the increase in e‐cigarette prevalence 12 and significant changes in the tobacco control climate 13. Thus, it is important to assess whether or not associations have remained stable and to obtain up‐to‐date effect sizes for use by policymakers. This study also includes affordability of smoking as a key potential confounder and assessment of cigarette consumption to address the question of whether e‐cigarettes are contributing to smoking reduction in people who continue to smoke.

This study addresses the following questions:

1. What is the association between prevalence of e‐cigarette use in current smokers and (a) prevalence of quit attempts among last‐year smokers, (b) overall quit rates among last‐year smokers and (c) mean cigarette consumption per day among current smokers?
2. What is the association between prevalence of e‐cigarette use during a quit attempt among last‐year smokers and (a) quit success rate among last‐year smokers and (b) overall quit rates among last‐year smokers?

## Methods

The protocol was pre‐registered on the Open Science Framework (https://osf.io/cpd7q/). An amendment was made to the protocol in December 2018. It was brought to our attention that, although price was accounted for in the original analysis by differencing, a more relevant variable which may explain the significant association with quit success rate may be the affordability of tobacco. The official affordability index based purely on the price of cigarettes started to show a decline approximately 1–2 years (in 2010) prior to the rise in use of e‐cigarettes, so would be unlikely to account for any association between e‐cigarette use and quitting 14. Moreover, smokers use price mitigation strategies to reduce the cost of smoking, most notably a reduction in daily cigarette consumption and a switch to cheaper cigarettes and hand‐rolled tobacco, and this means that true affordability may not be adequately captured using the retail price of cigarettes 15. The Smoking Toolkit Study (STS) collected data on smokers’ reports of the actual weekly cost of smoking, so we used this to derive a quarterly affordability index that bore a closer resemblance to what smokers were paying.

An additional amendment was made to the protocol in July 2019 following reviewer comments. E‐cigarette prevalence was estimated to be approximately 0.1% pre‐July 2009, when questions were first introduced into the STS 16, 17. This was judged to be reasonable, because although e‐cigarettes entered the market in England in 2005 they did not experience a significant uptake until 2011. Including this period from 2006 until 2009 into the model provides additional information in terms of the pattern of quitting activity when e‐cigarettes were not being used. The longer series also ultimately leads to a more accurate model 11. However, as a validity check, and as it is conceivable that associations may change over time, the primary unadjusted analyses were re‐run using data from the third quarter of 2009.

## Design

Data on the explanatory variables and outcome variables come from the STS, which is a survey of a representative sample of the population in England aged 16+ 18. It has been collecting data on smoking patterns among smokers and recent ex‐smokers since November 2006. Questions on the use of e‐cigarettes among smokers were introduced in May 2011 and on aids to a quit attempt in July 2009. The STS involves monthly household surveys using a random location sampling design, with initial random selection of grouped output areas (containing ~ 300 households), stratified by socio‐demographic characteristic and region. Interviewers then select houses within these areas that are most likely to fulfil quotas, and conduct face‐to‐face computer‐assisted interviews with one member per household. The quotas are tailored to the output area and the probability of certain groups being at home. Participants from the STS appear to be representative of the population in England, having similar socio‐demographic composition to other large national surveys, such as the Health Survey for England 18.

Data on the covariate mass media expenditure were obtained from Public Health England and the affordability index calculated using data from the Office for National Statistics and the STS.

## Measures

### Data on explanatory variables

Participants who reported that they smoked cigarettes (including hand‐rolled) every day or not every day were asked the following questions:

1. Which, if any, of the following are you currently using to help you cut down the amount you smoke? Response options: nicotine gum, nicotine replacement lozenges\tablets, nicotine replacement inhaler, nicotine replacement nasal spray, nicotine patch, electronic cigarette, nicotine mouth spray, other.
2. Do you regularly use any of the following in situations when you are not allowed to smoke? Response options: nicotine gum, nicotine replacement lozenges/tablets, nicotine replacement inhaler, nicotine replacement nasal spray, nicotine patch, electronic cigarette, nicotine mouth spray, other.
3. Can I check, are you using any of the following either to help you stop smoking, to help you cut down or for any other reason at all? Response options: nicotine gum, nicotine replacement lozenges\tablets, nicotine replacement inhaler, nicotine replacement nasal spray, nicotine patch, electronic cigarette, nicotine mouth spray, other.

Prevalence of use of e‐cigarettes in current smokers was obtained for each quarter by counting the number of respondents who answered ‘electronic cigarette’ in response to any of the three questions above, divided by the number of cigarette smokers.

Past‐year smokers who had made a quit attempt during the previous 12 months were asked the following question: ‘Which, if any, of the following did you try to help you stop smoking during the most recent serious quit attempt?’. Past‐year smokers responded by selecting from a list of cessation aids (including e‐cigarette use). Prevalence of use of e‐cigarettes in a quit attempt was calculated for each quarter by dividing the number of respondents who reported having used e‐cigarettes by the number of those who reported having made a quit attempt.

### Data on outcome variables

Smokers and last‐year were asked:

1. How many cigarettes per day do you usually smoke?
2. How many serious attempts to stop smoking have you made in the last 12 months? By serious attempt I mean you decided that you would try to make sure you never smoked again. Please include any attempt that you are currently making and please include any successful attempt made within the last year.
3. How long did your most recent serious quit attempt last before you went back to smoking?

The average cigarette consumption in each quarter was calculated as the total cigarette consumption each quarter divided by the number of respondents who reported that they were current smokers. The prevalence of quit attempts in each quarter was calculated as the number of respondents who report having made one or more quit attempt in the past 12 months divided by the number of past‐year smokers. The quit success rate in each quarter was calculated as the number of respondents reporting that they are still not smoking divided by the number reporting having made a quit attempt. The overall quit rate in each quarter was calculated as the proportion of past‐year smokers who report they are still not smoking following a quit attempt, and in sensitivity analyses as the proportion of past‐year smokers who reported that they stopped smoking in the past year.

### Data on covariates

In England, tobacco mass media campaigns have been run as part of a national tobacco control programme. Spending was almost completely suspended in 2010, and then re‐introduced in 2011 at a much lower level. Previous studies have shown that such cuts were associated with a decreased use of smoking cessation support 19, 20. Thus, advertising expenditure was adjusted for.

A number of tobacco control policies were adjusted for in the analyses assuming a simple 1‐month temporary pulse effect. These included the move in commissioning of stop smoking services from the National Health Service (NHS) to local authorities (326 organizations responsible for public services and facilities in a particular district of England) in April 2013 21, the introduction of a smoking ban in July 2007 22, licensing of nicotine replacement therapy (NRT) for harm reduction in December 2009 23, the publication of National Institute for Health and Care Excellence (NICE) guidance on harm reduction in June 2013 24, a change in the minimum age of sale of cigarettes October 2007 25 and the tobacco products directive in May 2016 26.

The affordability index was derived using a formula and data published by the Office for National Statistics 14:

Affordability of tobacco index=(adjusted real households’disposable income index/relative tobacco price index)×100,

$$\text{where relative tobacco price index}=\left(\text{tobacco price index}/\text{retail price index}\right)\times 100.$$

The adjusted real households’ disposable income index is an index of total households’ income, minus payments of income tax and other taxes, etc. per capita. The tobacco price index shows how much the average price of tobacco has changed compared with the price in the base year (set at 1987), while the retail price index shows by how much the prices of all items have changed compared with the price in the base year.

We replaced the tobacco price index with a weekly tobacco expenditure index derived from the STS 14, 18. Expenditure on smoking among current smokers was assessed by asking: ‘On average about how much per week do you think you spend on cigarettes or tobacco?’, and to report the number of cigarettes they smoked per day. Smokers’ average cost of smoking (in £/week) was derived from the following liberal assumptions for upper and lower estimates of plausible levels of consumption and expenditure per week: (1) smokers smoke a maximum of 560 cigarettes per week; (2) spending does not exceed £280 per week and (3) single cigarettes cost between £0.05 and £1 27. We also used a quarterly rather than yearly retail price index (adopted by the Office for National Statistics) and disposable income index, and changed the base year from 1987 to January 2007 as the start of our time–series analysis. Thus, an affordability index greater than 100 in a given quarter signifies that cigarettes were more affordable than in the first quarter of 2007.

## Analysis

All data were analysed in R 28. Strengthening the Reporting of Observational Studies in Epidemiology (STROBE) guidelines were followed throughout 29. Data were aggregated quarterly and were weighted to match the population profile in England on age, social grade, region, tenure, ethnicity and working status within sex. The dimensions are derived from a combination of the 2011 https://www.sciencedirect.com/topics/medicine-and-dentistry/population-research, Office for National Statistics and an annual random probability survey conducted for the National Readership Survey.

### Missing data

Data were only available on the prevalence of use of e‐cigarettes among smokers from April 2011, although data concerning use during a recent quit attempt were available from July 2009. Thus, the prevalence of e‐cigarette use among smokers between July 2009 and April 2011 was estimated from data on use during a quit attempt; use of e‐cigarettes among smokers between November 2006 and June 2009 was assumed to be 0.1% of smokers based on other surveys, which found their use to be extremely rare before 2009 16, 17.

Two waves of data were collected in March 2007, so these were combined. No data were collected in December 2008. Mean cigarette consumption and e‐cigarette use during this period were calculated as an average of the month before and the month after. For a few months (May 2012, July 2012, September 2012, November 2012, January 2013, March 2013), data on e‐cigarettes use among smokers were not recorded. For these months, the average of the previous and next month were imputed.

For a number of months, mass media spending was effectively zero and was imputed as 0.1 to allow the analysis to run, as data were log‐transformed to stabilize the variance. The same assumption was made for e‐cigarette use where prevalence in the sample was zero.

### Autoregressive Integrated Moving Average with Exogeneous Input (ARIMAX) modelling

ARIMAX analysis was used with the TSA package 30 to estimate the association of e‐cigarette use on cigarette consumption and quitting activity 31, 32, 33. ARIMAX is an extension of autoregressive integrated moving average analysis (ARIMA), which produces forecasts based upon prior values in the time–series analysis (AR terms) and the errors made by previous predictions (MA terms). Both adjusted and unadjusted models are reported in this paper.

Standard recommended procedures were used to select the ARIMAX models 31, 34. First, we assessed each time–series analysis for outlying values which may bias the results and the presence of exogeneity using the Granger causality test. No outliers were detected for any of the time–series analyses. The assumption of weak exogeneity (i.e. *Y* can depend on the lagged values of *X*, but the reverse must not be true) was met for all analyses except those involving daily cigarette consumption. As caution has been advised when using this test on data during a long time‐period, it was assumed that the association was due to chance 35, 36. Secondly, plots of the differenced data and unit root tests [i.e. Osborn–Chui–Smith–Birchenhall test (OCBS) and Kwiatkowski–Phillips–Schmidt–Shin (KPSS) test] were used to determine the number of seasonal and non‐seasonal differences required for the time–series analyses to be made stationary 37. This was confirmed by the augmented Dickey–Fuller test (ADF).

To identify the most appropriate transfer function for the continuous explanatory variables (i.e. to identify the manner in which past values of the e‐cigarette time–series analysis are used to forecast future values of the outcome), the sample cross‐correlation function was checked for each ARIMAX model, with pre‐whitened data 33. Pre‐whitening removes autocorrelation in the input series that may cause spurious cross‐correlation effects. Additional checks were also run by comparing univariate ARIMAX models with variations for the transfer function.

Next, in order to determine the initial values of the AR and MA terms for the baseline models, the autocorrelation function (ACF) and partial autocorrelation function (PACF) were assessed. Additional models with various fitted AR and MA terms were then compared to this baseline model using the Akaike information criterion (AIC). According to the Box–Jenkins method, in ARIMA (p, d, q) the values of p and q should be 2 or less, or the total number of parameters should be less than 3 31. Therefore, we only checked ARIMAX models for p and q values of 3 or fewer. The models with lower AIC values were selected.

Finally, the ACF for the residuals of the best‐fitting models were checked for additional correlation (thus the need for additional MA/AR seasonal or non‐seasonal terms), and the coefficients of the correlation terms assessed for significance and whether they fall within the bounds of stationarity and invertibility 38. The Ljung–Box test for white noise and plot of the ACF for model residuals were also used to statistically evaluate the degree to which the residuals are free of serial correlation 39, and the final model residuals assessed for normality.

## Results

### Sample characteristics

Data were collected on 222 856 adults aged 16 years and over taking part in the STS who reported their smoking status. Of these, 20.27% weighted [95% confidence interval (CI) weighted = 20.11–20.44; *n* = 45 173] were current smokers and 22.30% weighted (95% CI weighted = 22.14–22.48; *n* = 50 498) were past‐year smokers.

### Association between e‐cigarette use among current smokers and quit attempts among past‐year smokers

**Figure 1:**
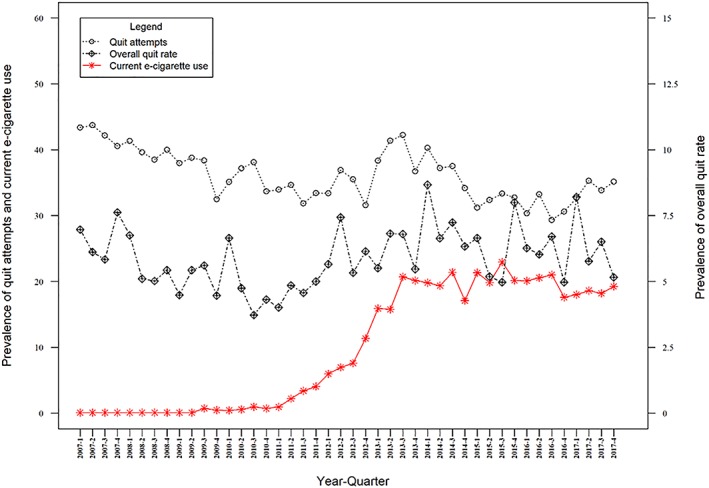
Quarterly prevalence of quit attempt rate, overall quit rate and current use of e‐cigarettes in England [Colour figure can be viewed at http://wileyonlinelibrary.com]

In adjusted and unadjusted analyses, the data showed no clear association between prevalence of e‐cigarette use among current smokers and attempts to quit smoking (B = 0.011, 95% CI = –0.046 to 0.069, *P* = 0.698; see Table 1 and Fig. 1 and Supporting information, Table S1).

**Table 1:** Adjusted estimated percentage point changes in quitting activities and cigarette consumption as a function of current e‐cigarette use, based on ARIMAX models.

	Quit attempts			Overall quit rate			Average cigarette consumption per day		
	Percentage change per 1% change in the exposure	95% CI	*P*‐value	Percentage change per 1% change in the exposure	95% CI	*P*‐value	Percentage change per 1% change in the exposure	95% CI	*P*‐value
Prevalence of current e‐cigarette use	0.011	–0.046 to 0.069	0.698	0.054	0.032 to 0.076	< 0.001	0.019	–0.043 to 0.082	0.542
Mass media	0.026	−0.014 to 0.066	0.196	0.214	0.118 to 0.309	< 0.001	−0.005	−0.032 to 0.023	0.739

	Total change due to the exposure	95% CI	P‐value	Total change due to the exposure	95% CI	P‐value	Total change due to the exposure	95% CI	P‐value
Smoking ban (temporary impact in third quarter of 2007)	−0.026	−0.133 to 0.081	0.636	0.480	0.187 to 0.772	0.001	−0.034	−0.084 to 0.017	0.193
Increase in age of sale (temporary impact in fourth quarter of 2007)	0.036	−0.070 to 0.143	0.503	0.446	0.147 to 0.745	0.003	0.011	−0.041 to 0.064	0.674
Move to local authority (temporary impact in second quarter of 2013)	0.041	−0.055 to 0.138	0.403	−0.027	−0.320 to 0.265	0.854	0.018	−0.034 to 0.071	0.498
Tobacco control directive (temporary impact in the second quarter of 2016)	−0.078	−0.169 to 0.012	0.089	0.147	−0.143 to 0.436	0.322	−0.039	−0.106 to 0.029	0.261
Model Lag for e‐cigarettes Lag for mass media Adjusted *R* ^2^	ARIMA(0,1,0)(1,0,0)_4_ No lag No lag 65.47			ARIMA(0,1,1)(1,0,0)_4_ No lag No lag 45.97			ARIMA(0,1,1)(1,0,0)_4_ No lag No lag 92.34		

ARIMA = autoregressive integrated moving average analysis; ARIMAX = autoregressive integrated moving average with exogeneous input; CI = confidence interval.

### Association between e‐cigarette use among current smokers and cigarette consumption among current smokers

In adjusted and unadjusted analyses, the data did not show a clear association between prevalence of e‐cigarette use among current smokers and cigarette consumption (BAdj = 0.019 95% CI = –0.043 to 0.082, *P* = 0.542; see Table 1 and Fig. 3 and Supporting information, Table S1).

### Association between e‐cigarette use during a quit attempt and quit success rate among past‐year smokers

**Figure 2:**
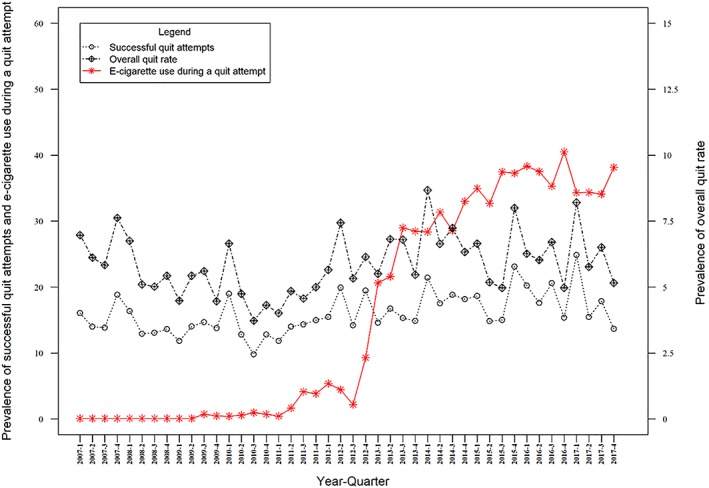
Quarterly prevalence of quit success rate, overall quit rate and use of e‐cigarettes in a quit attempt England [Colour figure can be viewed at http://wileyonlinelibrary.com]

Prevalence of e‐cigarette use during a quit attempt was positively associated with the quit success rate, with every 1% rise in use associated with a 0.060% increase in the quit success rate. In addition, there was evidence of an increase in the quit success rate following the increase in the age of sale of cigarettes and the smoking ban, and a positive association with mass media spending (see Table 2 and Fig. 2 and Supporting information, Table S2). Table 3

**Table 2:** Adjusted estimated percentage point changes in quitting activities and cigarette consumption as a function of use of e‐cigarettes during a quit attempt, based on ARIMAX models.

	Quit success rate			Overall quit rate		
	Percentage change per 1% change in the exposure	95% CI	*P*‐value	Percentage change per 1% change in the exposure	95% CI	*P*‐value
Prevalence of e‐cigarette use during a quit attempt	0.060	0.043 to 0.078	< 0.001	0.050	0.031 to 0.069	< 0.001
Mass media	0.143	0.055 to 0.231	0.001	0.212	0.1179 to 0.305	< 0.001

	Total change due to the exposure	95% CI	P‐value	Total change due to the exposure	95% CI	P‐value
Smoking ban (temporary impact in third quarter of 2007)	0.400	0.126 to 0.674	0.004	0.472	0.186 to 0.759	0.001
Increase in age of sale (temporary impact in fourth quarter of 2007)	0.318	0.041 to 0.596	0.025	0.439	0.147 to 0.731	0.003
Move to local authority (temporary impact in second quarter of 2013)	−0.216	−0.489 to 0.056	0.120	−0.020	−0.308 to 0.269	0.895
Tobacco control directive (temporary impact in the second quarter of 2016)	0.181	−0.091 to 0.453	0.192	0.130	−0.157 to 0.417	0.375
Model Lag for e‐cigarettes Lag for mass media Adjusted *R* ^2^	ARIMA(0,1,1)(1,0,0)_4_ No lag No lag 48.61			ARIMA(0,1,1)(1,0,0)_4_ No lag No lag 47.47		

ARIMA = autoregressive integrated moving average analysis; ARIMAX = Autoregressive Integrated Moving Average with Exogeneous Input; CI = confidence interval.

**Table 3:** Adjusted estimated percentage point changes in overall quit rate as a function of current e‐cigarette use, based on ARIMAX models including the affordability index.

	Overall quit rate		
	Percentage change per 1% change in the exposure	95% CI	*P*‐value
Prevalence of current e‐cigarette use	0.036	−0.001 to 0.074	0.057
Mass media Affordability	0.104 0.004	0.059 to 0.148 −0.007 to 0.015	< 0.001 0.467

	Total change due to the exposure	95% CI	P‐value
Smoking ban (temporary impact in third quarter of 2007)	0.397	0.105 to 0.688	0.008
Increase in age of sale (temporary impact in fourth quarter of 2007)	0.481	0.180 to 0.783	0.002
Move to local authority (temporary impact in second quarter of 2013)	−0.015	−0.307 to 0.277	0.919
Tobacco control directive (temporary impact in the second quarter of 2016)	0.168	−0.142 to 0.477	0.289
Model Lag for e‐cigarettes Lag for mass media Lag for affordability Adjusted *R* ^2^	ARIMA(0,1,1)(1,0,0)_4_ No lag No lag No lag 0.48		

ARIMA = autoregressive integrated moving average analysis; ARIMAX = Autoregressive Integrated Moving Average with Exogeneous Input; CI = confidence interval.

### Association between e‐cigarette use during a quit attempt and current e‐cigarette use with overall quit rate among past‐year smokers

In adjusted analyses, prevalence of e‐cigarette use during a quit attempt was positively associated with the overall quit rate, with every 1% rise in use associated with a 0.050% increase in the overall quit rate. Prevalence of e‐cigarette use in current smokers was also positively associated with the overall quit rate, such that for every 1% increase in e‐cigarette use, the overall quit rate increased by 0.054%. In addition, there was evidence of a rise in the overall quit rate following the increase in the age of sale of cigarettes and the smoking ban, and a positive association between mass media spending and the overall quit rate. In the unadjusted analyses, the data did not show clear associations between the explanatory e‐cigarette variables and the overall quit rate (see Tables 1, 2, Figs 1, 2 and Supporting information, Table S1).

**Figure 3:**
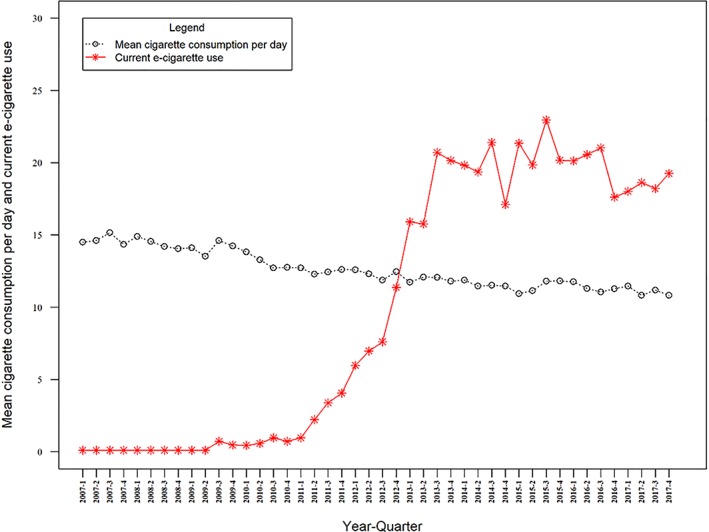
Quarterly prevalence of mean daily cigarette consumption and current use of e‐cigarettes in England [Colour figure can be viewed at http://wileyonlinelibrary.com]

## Sensitivity analyses

### Association between e‐cigarette use among current smokers and quit success rate

In adjusted analyses, prevalence of e‐cigarette use among current smokers was positively associated with the quit success rate, such that for every 1% increase in e‐cigarette use, the quit success rate increased by 0.066%. In the unadjusted analysis, the data did not show a clear association (see Supporting information, Fig. S1, Table S2).

### Redefining overall quit rates

A prevalence of 7.00% [95% CI = 6.63–7.37; standard deviation (SD) = 1.26] was found for overall quit rates when redefining it as the proportion of past‐year smokers who had stopped in the past year. In adjusted analyses, prevalence of e‐cigarette use among current smokers was positively associated with the redefined overall quit rate, such that for every 1% increase in e‐cigarette use, the redefined overall quit rate increased by 0.044%. Prevalence of e‐cigarette use during a quit attempt was also positively associated with the redefined overall quit rate, with every 1% rise in use associated with a 0.041% increase in the redefined overall quit rate. In addition, there was evidence of a rise following the increase in the age of sale of cigarettes and the smoking ban, and a positive association between mass media spending and the redefined overall quit rate. In the unadjusted analyses, the data did not show a clear association between the explanatory e‐cigarette variables and the redefined overall quit rate (see Supporting information, Figs S2, S3, Table S3).

### Adjustment for affordability

After adjusting for affordability, associations were still present between prevalence of e‐cigarette use during a quit attempt and quit success rate and overall quit rates (see Table 4 and Supporting information, Table S4, Fig. S4). They were also still present between current e‐cigarette use and overall quit rates (see Table 3)

**Table 4:** Adjusted estimated percentage point changes in quit success rate and overall quit rate as a function of e‐cigarette use during a quit attempt, based on ARIMAX models including the affordability index.

	Quit success rate			Overall quit rate		
	Percentage change per 1% change in the exposure	95% CI	*P*‐value	Percentage change per 1% change in the exposure	95% CI	*P*‐value
Prevalence of e‐cigarette use during a quit attempt	0.062	0.028 to 0.096	< 0.001	0.085	0.035 to 0.135	0.001
Mass media Affordability	0.072 −0.002	0.031 to 0.113 −0.013 to 0.009	0.001 0.754	0.066 −0.018	−0.001 to 0.124 −0.033 to −0.002	0.055 0.023

	Total change due to the exposure	95% CI	P‐value	Total change due to the exposure	95% CI	P‐value
Smoking ban (temporary impact in third quarter of 2007)	0.363	0.090 to 0.636	0.009	0.442	0.039 to 0.855	0.036
Increase in age of sale (temporary impact in fourth quarter of 2007)	0.325	0.048 to 0.603	0.021	0.265	−0.173 to 0.702	0.235
Move to local authority (temporary impact in second quarter of 2013)	−0.238	−0.508 to 0.033	0.085	−0.143	−0.564 to 0.278	0.506
Tobacco control directive (temporary impact in the second quarter of 2016)	0.226	−0.055 to 0.507	0.115	0.425	0.011 to 0.838	0.044
Model Lag for e‐cigarettes Lag for mass media Lag for affordability Adjusted *R* ^2^	ARIMA(0,1,1)(1,0,0)_4_ None None None 0.51			ARIMA(0,1,1)(1,0,0)_4_ None None None 0.39		

ARIMA = autoregressive integrated moving average analysis; ARIMAX = Autoregressive Integrated Moving Average with Exogeneous Input; CI = confidence interval.

Restricting the analysis to the third quarter of 2009 until the fourth quarter of 2010

A positive association between current e‐cigarette prevalence and prevalence of e‐cigarette use during a quit attempt with quit success rate and overall quit rate was still established with the shorter time–series. The effect sizes were substantially larger than for the primary analysis. In addition, a significant negative association emerged between current e‐cigarette prevalence and average cigarette consumption per day, such that every 1% rise in use of e‐cigarettes was associated with a −0.037% decrease in average cigarette consumption (see Supporting information, Table S5).

## Discussion

### Principal findings

The increase in prevalence of e‐cigarette use in England was positively associated with the success rates of quit attempts and overall quit rates after adjustment for a range of confounding variables. No clear association was found between e‐cigarette use and prevalence of quit attempts or mean daily cigarette consumption.

### Study limitations

This study had several limitations. First, the STS required participants to recall use of smoking cessation aids during the previous 12 months; this could have introduced bias, although we have no reason to believe that reporting would differ over time. Secondly, although this study found evidence for associations between the change in age of sale and the smoking ban with quitting activities, only pulse effects for the tobacco control policies, i.e. an immediate temporary effect, were assessed. Future studies should consider variations in policy effects, such as more prolonged pulse effects, delayed effects and sustained effects 31. Thirdly, the findings might not generalize to other countries. England has a strong tobacco control climate and generally high motivation to quit among smokers, and a relatively liberal regulatory framework for e‐cigarettes. In countries with weaker tobacco control or stricter regulation of e‐cigarettes, different effects may be observed. Fourthly, although we are unaware of any other major population‐level intervention or other events during the study period, residual confounding cannot be ruled out. Smokers’ characteristics were not included in the study for several reasons: first, as the prevalence of demographic characteristics among smokers does not vary substantially over time and secondly, at a population‐level, age and social grade do not appear to be strongly associated with quit success 40. Fifthly, caution should be taken when interpreting null effects, and readers should not assume that they represent no association 41, 42. However, this study was powered to detect relatively small associations (see sample size calculation in 3), with enough data to adequately adjust for the number of confounding variables, seasonality and autocorrelation 11. Therefore, we can be confident that large associations do not exist between prevalence of e‐cigarette use, quit attempt prevalence and average cigarette consumption. Finally, for a full assessment of the impact of e‐cigarettes on population health, future studies should assess the impact on use among never smokers. Although previous studies report a rise in experimentation by never smokers, regular use remains rare in England 43, 44.

### Comparison with other studies

The findings on smoking cessation confirm those of previous time–series analyses in England and a subsequent population‐level study of e‐cigarette use among US adult smokers showing a significant increase in smoking cessation rates among e‐cigarette users 3, 45. They contradict claims that e‐cigarettes, as used by smokers in the population, undermine smoking cessation 1, 46, 47, 48.

The primary findings on cigarette consumption conflict with experimental studies showing large decreases in consumption when smokers start using e‐cigarettes 6. It is possible that, without the structure of a randomized controlled trial, use of e‐cigarettes to reduce smoking has a minimal impact overall. However, the sensitivity analysis restricting data to post‐July 2009 suggests that, in more recent years, e‐cigarette prevalence may be associated with a decline in average cigarette consumption. If this association is causal, it may be explained by an increase in dual use or use of e‐cigarettes for harm reduction, whereby smokers substitute some of their cigarettes with e‐cigarettes 49. Of course, caution should also be advised, as although there was no evidence of over‐parameterization with the shorter time–series, the ARIMAX is likely to have been less accurate 11. It will be important to continually monitor population‐level associations, as it is conceivable that these could change over time.

### Implications

If the association identified in the current study between increase in e‐cigarette use and the quit success rate is causal, then every 1 percentage point increase in e‐cigarette use in quit attempts would result in a 0.060 percentage point increase in quit success rate, other things being equal. Assuming that 34.3% of 7 million current smokers in 2017 were attempting to quit 50, 51, 52 and prevalence of e‐cigarette use in a quit attempt was 35.2% in that year 53, it is estimated that 845 152 (7 000 000 × 0.343 × 0.352) smokers used e‐cigarettes during a quit attempt; this equates to 50 700 (845 152 × 0.060) additional past‐year smokers who report that they are no longer smoking as a consequence of e‐cigarette use in a quit attempt in 2017. This is broadly similar to the estimate which we reported for 2015 3.

This estimate assumes that the use of e‐cigarettes to support a serious quit attempt is the primary mechanism by which e‐cigarettes help smokers transition to ex‐smokers. However, there may be other routes to stopping, such as experimentation with an e‐cigarette leading to quitting without an explicit attempt to stop 54.

If the association between change in current e‐cigarette use and overall quit rates is causal, then every 10 percentage point increase in current e‐cigarette use by current smokers would result in a 0.54 percentage point increase in overall quit rates, other things being equal. With 7 million current smokers in 2017 50, 51 and prevalence of current e‐cigarette use at 18.5% in that year 53 (7 000 000 × 0.185 × 0.054), this would equate to 69 930 additional past‐year smokers who report that they are no longer smoking as a consequence of e‐cigarettes in 2017.

One explanation for the difference in estimates for overall quit rates and quit success rate is that some smokers may quit without reporting having made a quit attempt. This would result in a higher estimate for the overall quit rate. Of course, the two estimates are also subject to margins of error both statistically and methodologically.

## Conclusions

The increase in prevalence of e‐cigarette use by smokers in England has been positively associated with an increase in success rates of quit attempts and overall quit rates, after adjusting for a range of possible confounding variables.

### Ethical approval

Ethical approval for the STS was granted by the UCL ethics committee (ID 0498/001). Ethical approval was not required for use of data from stop smoking services, as the data are publicly available. For access to the STS, please contact JB (jamie.brown@ucl.ac.uk).

### Declaration of interests

All authors have completed the ICMJE uniform disclosure form at http://www.icmje.org/coi_disclosure.pdf (available on request from the corresponding author) and declare that: R.W. undertakes consultancy and research for and receives travel funds and hospitality from manufacturers of smoking cessation medications but does not, and will not, take funds from e‐cigarettes manufacturers or the tobacco industry. R.W. is an adviser to the National Centre for Smoking Cessation and Training. R.W.'s salary is funded by Cancer Research UK. E.B. and J.B. have received unrestricted research funding from Pfizer; S.M., E.B. and J.B. are funded by CRUK (C1417/A14135); S.M. and E.B. receive funding from the SPHR; and J.B. is funded by the Society for the Study of Addiction. All authors declare there are no other relationships or activities that could appear to have influenced the submitted work.

### Acknowledgements

The STS is currently primarily funded by Cancer Research UK (C1417/A14135; C36048/A11654; C44576/A19501), and has previously also been funded by Pfizer, GlaxoSmithKline and the Department of Health. J.B.'s post is funded by a fellowship from the Society for the Study of Addiction, and Cancer Research UK also provide support (C1417/A14135); R.W. is funded by Cancer Research UK (C1417/A14135). E.B. is funded by a fellowship from the National Institute for Health Research's (NIHR) School for Public Health Research (SPHR) (SPHR‐SWP‐ALC‐WP5), and Cancer Research UK also provide support (C1417/A14135). S.W. is funded by Cancer Research UK (C1417/A14135), and the SPHR (SPHR‐SWP‐ALC‐WP5) also provide support. The SPHR is a partnership between the Universities of Sheffield, Bristol, Cambridge, and Exeter; University College London (UCL); London School for Hygiene and Tropical Medicine; LiLaC collaboration between the Universities of Liverpool and Lancaster and Fuse; and the Centre for Translational Research in Public Health, a collaboration between Newcastle, Durham, Northumbria, Sunderland and Teesside Universities. The views expressed are those of the authors and not necessarily those of the NHS, NIHR or Department of Health. No funders had any involvement in the design of the study, the analysis or interpretation of the data, the writing of the report or the decision to submit the paper for publication.

## Supporting information


**Table S1** Unadjusted estimated percentage point changes in quitting activities and cigarette consumption as a function of current e‐cigarette use and use during a quit attempt, based on autoregressive integrated moving average with exogeneous input (ARIMAX) models.
**Table S2** Unadjusted and adjusted estimated percentage point changes in quit success rate as a function of current e‐cigarette use, based on autoregressive integrated moving average with exogeneous input (ARIMAX) models.
**Table S3** Unadjusted and adjusted estimated percentage point changes in the redefined overall quit rates as a function of current e‐cigarette use and use during a quit attempt, based on autoregressive integrated moving average with exogeneous input (ARIMAX) models.
**Table 4** Unadjusted estimated percentage point changes in quitting activities as a function of affordability, based on autoregressive integrated moving average with exogeneous input (ARIMAX) models.
**Table 5** Unadjusted estimated percentage point changes in quitting activities and cigarette consumption as a function of current e‐cigarette use and use during a quit attempt, based on autoregressive integrated moving average with exogeneous input (ARIMAX) models (unadjusted) using data from July 2009.
**Figure S1** Quarterly prevalence of quit success rate and current use of e‐cigarettes in England.
**Figure S2** Quarterly prevalence of overall quit rate and current use of e‐cigarettes and in England.
**Figure S3** Quarterly prevalence of overall quit rate and use of e‐cigarettes during a quit attempt in England.
**Figure S4**Affordability index.
