# Supplementary material for: Association of prevalence of electronic cigarette use with smoking cessation and cigarette consumption in England: a time–series analysis between 2006 and 2017
Source: Addiction. 2019 Dec 4;115(5):961–74. doi: 10.1111/add.14851 (PMC7187187; doi:10.1111/add.14851)
Supplement: Supplementary file 1 — Table S1 Unadjusted estimated percentage point changes in quitting activities and cigarette consumption as a function of current e‐cigarette use and use during a quit attempt, based on autoregressive integrated moving average with exogeneous input (ARIMAX) models. Table S2 Unadjusted and adjusted estimated percentage point changes in quit success rate as a function of current e‐cigarette use, based on autoregressive integrated moving average with exogeneous input (ARIMAX) models. Table S3 Unadjusted and adjusted estimated percentage point changes in the redefined overall quit rates as a function of current e‐cigarette use and use during a quit attempt, based on autoregressive integrated moving average with exogeneous input (ARIMAX) models. Table 4 Unadjusted estimated percentage point changes in quitting activities as a function of affordability, based on autoregressive integrated moving average with exogeneous input (ARIMAX) models. Table 5 Unadjusted estimated percentage point changes in quitting activities and cigarette consumption as a function of current e‐cigarette use and use during a quit attempt, based on autoregressive integrated moving average with exogeneous input (ARIMAX) models (unadjusted) using data from July 2009. Figure S1 Quarterly prevalence of quit success rate and current use of e‐cigarettes in England. Figure S2 Quarterly prevalence of overall quit rate and current use of e‐cigarettes and in England. Figure S3 Quarterly prevalence of overall quit rate and use of e‐cigarettes during a quit attempt in England. Figure S4Affordability index. [file ADD-115-961-s001.docx]

**Supplementary Table 1:** Unadjusted estimated percentage point changes in quitting activities and cigarette consumption as a function of current e-cigarette use and use during a quit attempt, based on autoregressive integrated moving average with exogeneous input (ARIMAX) models

|  | Quit attempts | | | Quit success rate | | | Overall quit rate | | | Average cigarette consumption per day | | |
| --- | --- | --- | --- | --- | --- | --- | --- | --- | --- | --- | --- | --- |
|  | **Percentage change per 1% change in the exposure** | **95% CI** | **P value** | **Percentage change per 1% change in the exposure** | **95% CI** | **P value** | **Percentage change per 1% change in the exposure** | **95% CI** | **P value** | **Percentage change per 1% change in the exposure** | **95% CI** | **P value** |
| Prevalence of current e-cigarette use | 0.018 | -0.043 to 0.078 | 0.568 | NA |  |  | 0.031 | -0.056 to 0.117 | 0.485 | -0.001 | -0.035 to 0.034 | 0.966 |
| Model  Lag  Adjusted R^2^ | ARIMA(0,1,0)(1,0,0)_4_  No lag  61.97 | | |  | | | ARIMA(0,1,1)(1,0,0)_4_  No lag  15.31 | | | ARIMA(0,1,1)(1,0,0)_4_  No lag  91.30 | | |
| Prevalence of e-cigarette use during a quit attempt | NA |  |  | 0.042 | 0.022 to 0.062 | <0.001 | 0.029 | -0.046 to 0.104 | 0.453 | NA |  |  |
| Model  Lag  Adjusted R^2^ |  | | | ARIMA(0,1,1)(1,0,0)_4_  No lag  33.09 | | | ARIMA(0,1,1)(1,0,0)_4_  No lag  15.68 | | |  | | |

**Supplementary Table 2:** Unadjusted and adjusted estimated percentage point changes in quit success rate as a function of current e-cigarette use, based on autoregressive integrated moving average with exogeneous input (ARIMAX) models

|  | **Unadjusted**  Quit success rate | | | **Adjusted**  Quit success rate | | |
| --- | --- | --- | --- | --- | --- | --- |
|  | **Percentage change per 1% change in the exposure** | **95% CI** | **P value** | **Percentage change per 1% change in the exposure** | **95% CI** | **P value** |
| Prevalence of current e-cigarette use | 0.038 | -0.009 to 0.086 | 0.116 | 0.066 | 0.046 to 0.86 | <0.001 |
| *Mass media* |  |  |  | 0.147 | 0.056 to 0.237 | 0.002 |
|  | **Total change due to the exposure** | **95% CI** | **P value** | **Total change due to the exposure** | **95% CI** | **P value** |
| *Smoking ban (temporary impact in third quarter of 2007)* |  |  |  | **0.412** | **0.132 to 0.692** | **0.004** |
| *Increase in age of sale (temporary impact in fourth quarter of 2007)* |  |  |  | **0.333** | **0.048 to 0.618** | **0.022** |
| *Move to local authority (temporary impact in second quarter of 2013)* |  |  |  | **-0.229** | **-0.511 to 0.052** | **0.110** |
| *Tobacco control directive (temporary impact in the second quarter of 2016)* |  |  |  | **0.199** | **-0.077 to 0.475** | **0.157** |
| Model  Lag for e-cigarettes  Lag for mass media  Adjusted R^2^ | ARIMA(0,1,1)(1,0,0)_4_  No lag  20.65 | | | ARIMA(0,1,1)(1,0,0)_4_  No lag  No lag  46.94 | | |

**Supplementary Table 3:** Unadjusted and adjusted estimated percentage point changes in the redefined overall quit rates as a function of current e-cigarette use and use during a quit attempt, based on autoregressive integrated moving average with exogeneous input (ARIMAX) models

|  | **Unadjusted**  Current e-cigarette use | | | **Adjusted**  Current e-cigarette use | | | **Unadjusted**  Use of e-cigarettes during a quit attempt | | | **Adjusted**  Use of e-cigarettes during a quit attempt | | |
| --- | --- | --- | --- | --- | --- | --- | --- | --- | --- | --- | --- | --- |
|  | **Percentage change per 1% change in the exposure** | **95% CI** | **P value** | **Percentage change per 1% change in the exposure** | **95% CI** | **P value** | **Percentage change per 1% change in the exposure** | **95% CI** | **P value** | **Percentage change per 1% change in the exposure** | **95% CI** | **P value** |
| E-cigarette | 0.017 | -0.010 to 0.044 | 0.223 | 0.044 | 0.024 to 0.065 | <0.001 | 0.017 | -0.008 to 0.041 | 0.182 | 0.041 | 0.023 to 0.059 | <0.001 |
| *Mass media* |  |  |  | 0.212 | 0.119 to 0.305 | <0.001 |  |  |  | 0.212 | 0.121 to 0.302 | <0.001 |
|  | **Total change due to the exposure** | **95% CI** | **P value** | **Total change due to the exposure** | **95% CI** | **P value** | **Total change due to the exposure** | **95% CI** | **P value** | **Total change due to the exposure** | **95% CI** | **P value** |
| *Smoking ban (temporary impact in third quarter of 2007)* |  |  |  | **0.443** | **0.158 to 0.728** | **0.002** |  |  |  | **0.441** | **0.161 to 0.721** | **0.002** |
| *Increase in age of sale (temporary impact in fourth quarter of 2007)* |  |  |  | **0.435** | **0.155 to 0.715** | **0.002** |  |  |  | **0.428** | **0.152 to 0.704** | **0.002** |
| *Move to local authority (temporary impact in second quarter of 2013)* |  |  |  | **0.060** | **-0.212 to 0.333** | **0.664** |  |  |  | **0.061** | **-0.208 to 0.330** | **0.658** |
| *Tobacco control directive (temporary impact in the second quarter of 2016)* |  |  |  | **0.199** | **-0.067 to 0.465** | **0.144** |  |  |  | **0.187** | **-0.077 to 0.452** | **0.165** |
| Model  Lag for e-cigarettes  Lag for mass media  Adjusted R^2^ | ARIMA(0,0,1)(0,0,0)_4_  No lag  55.34 | | | ARIMA(0,0,1)(0,0,0)_4_  No lag  No lag  47.19 | | | ARIMA(0,0,1)(0,0,0)_4_  No lag  58.41 | | | ARIMA(0,0,1)(0,0,0)_4_  No lag  No lag  47.94 | | |

**Supplementary Table 4:** Unadjusted estimated percentage point changes in quitting activities as a function of affordability, based on autoregressive integrated moving average with exogeneous input (ARIMAX) models

|  | Quit success rate | | | | | Overall quit rate | | | | |
| --- | --- | --- | --- | --- | --- | --- | --- | --- | --- | --- |
|  | **Percentage change per 1% change in the exposure** | | **95% CI** | **P value** | | **Percentage change per 1% change in the exposure** | | **95% CI** | | **P value** |
| Affordability | -0.195 | -1.779 to 1.389 | | | 0.809 | -0.229 | -1.707 to 1.248 | | 0.761 | |
| Model overall  Lag overall  Adjusted R^2^ overall | ARIMA(0,1,1)(1,0,0)_4_  No lag  18.98 | | | | | ARIMA(0,1,1)(1,0,0)_4_  No lag  15.84 | | | | |

**Supplementary Table 5:** Unadjusted estimated percentage point changes in quitting activities and cigarette consumption as a function of current e-cigarette use and use during a quit attempt, based on autoregressive integrated moving average with exogeneous input (ARIMAX) models (unadjusted) using data from July 2009

|  | Quit attempts | | | Quit success rate | | | Overall quit rate | | | Average cigarette consumption per day | | |
| --- | --- | --- | --- | --- | --- | --- | --- | --- | --- | --- | --- | --- |
|  | **Percentage change per 1% change in the exposure** | **95% CI** | **P value** | **Percentage change per 1% change in the exposure** | **95% CI** | **P value** | **Percentage change per 1% change in the exposure** | **95% CI** | **P value** | **Percentage change per 1% change in the exposure** | **95% CI** | **P value** |
| Prevalence of current e-cigarette use | 0.033 | -0.081 to 0.147 | 0.572 | 0.083 | 0.041 to 0.126 | <0.001 | 0.095 | 0.053 to 0.136 | <0.001 | -0.037 | -0.065 to -0.009 | <0.001 |
| Model  Lag | ARIMA(0,1,0)(1,0,0)_4_  No lag | | |  | | | ARIMA(0,1,1)(1,0,0)_4_  No lag | | | ARIMA(0,1,1)(1,0,0)_4_  No lag | | |
| Prevalence of e-cigarette use during a quit attempt | NA |  |  | 0.069 | 0.035 to 0.102 | <0.001 | 0.075 | 0.040 to 0.110 | <0.001 | NA |  |  |
| Model  Lag |  | | | ARIMA(0,1,1)(1,0,0)_4_  No lag | | | ARIMA(0,1,1)(1,0,0)_4_  No lag | | |  | | |


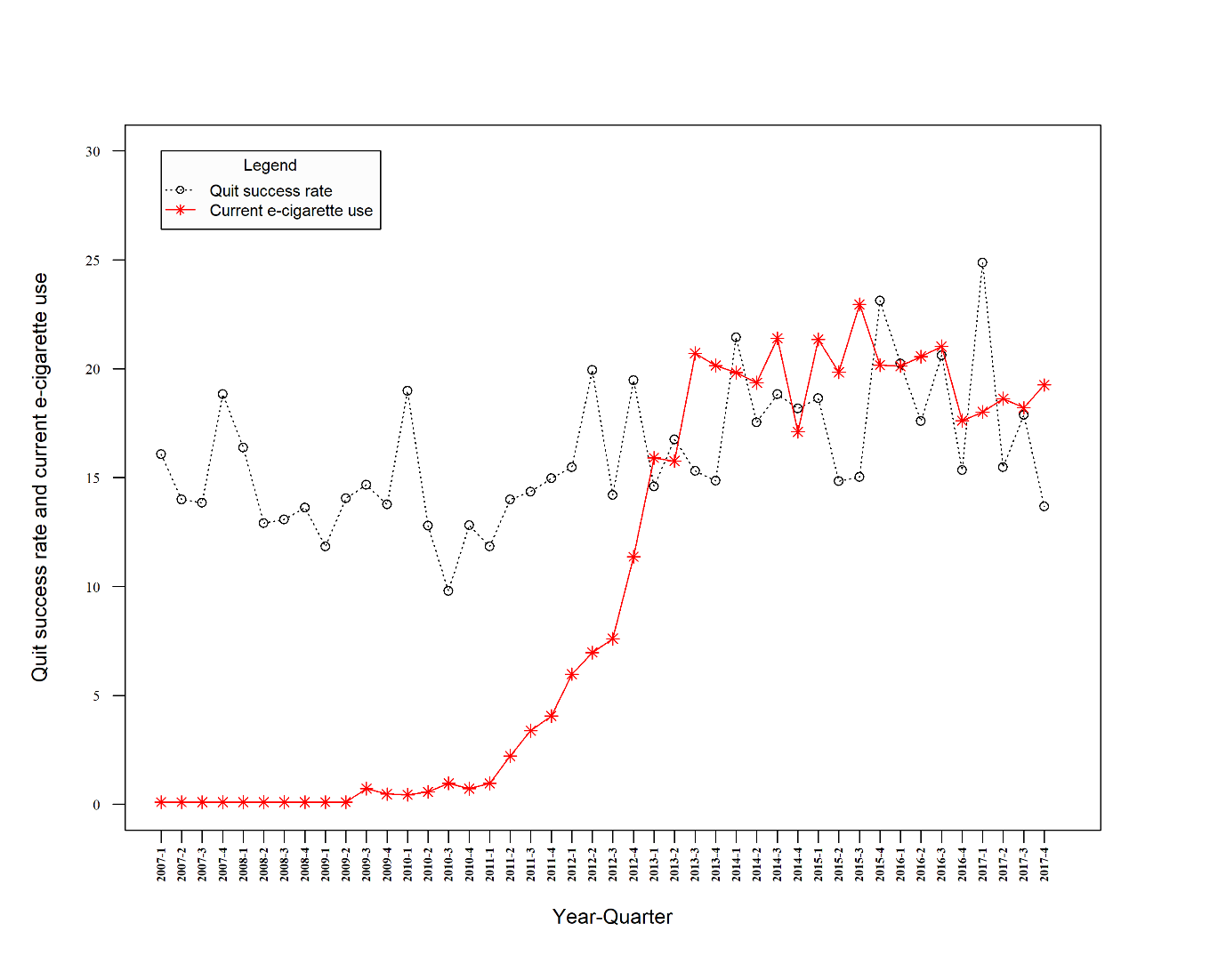


**Supplementary Figure 1:** Quarterly prevalence of quit success rate and current use of e-cigarettes in England


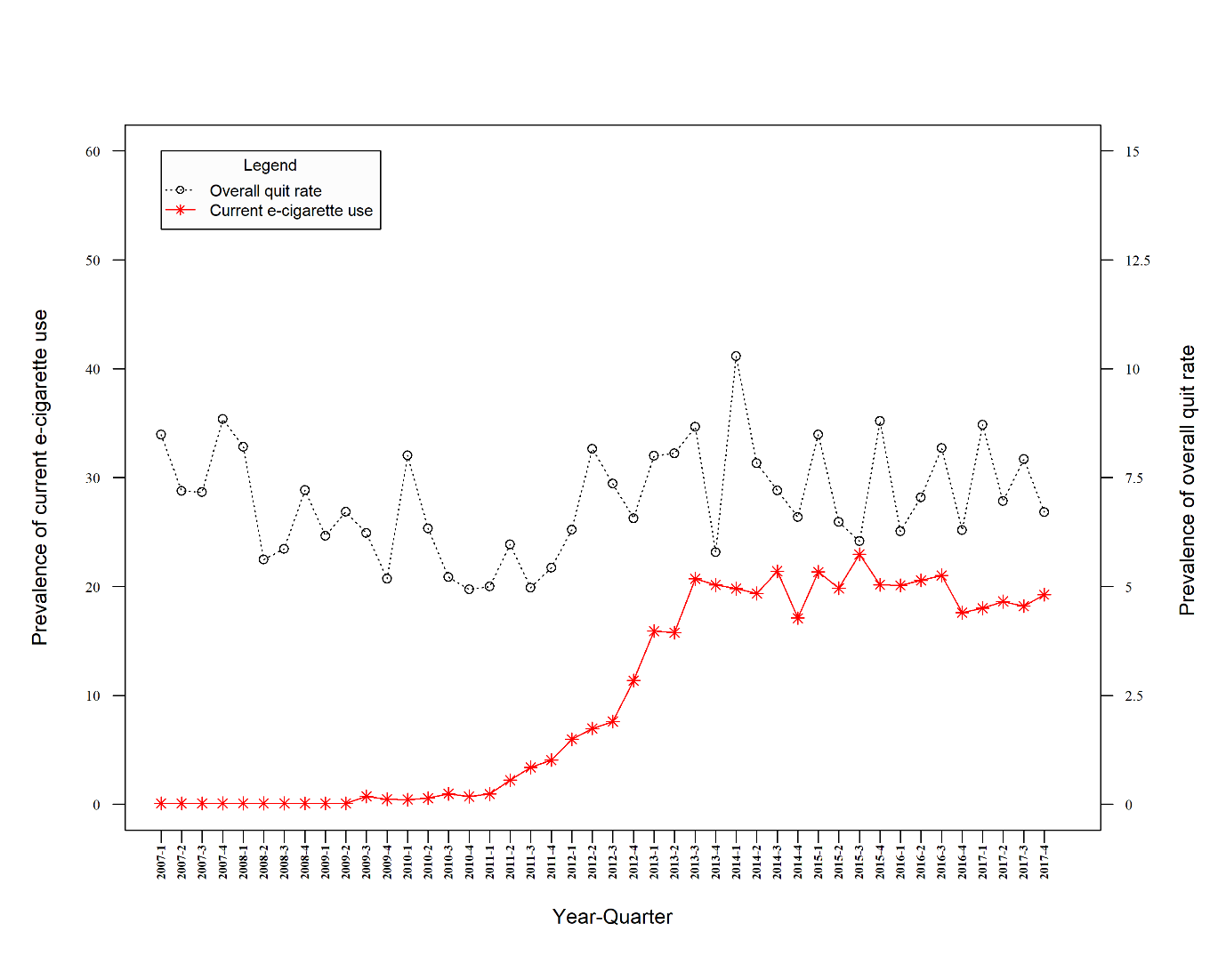


**Supplementary Figure 2:** Quarterly prevalence of overall quit rate and current use of e-cigarettes and in England


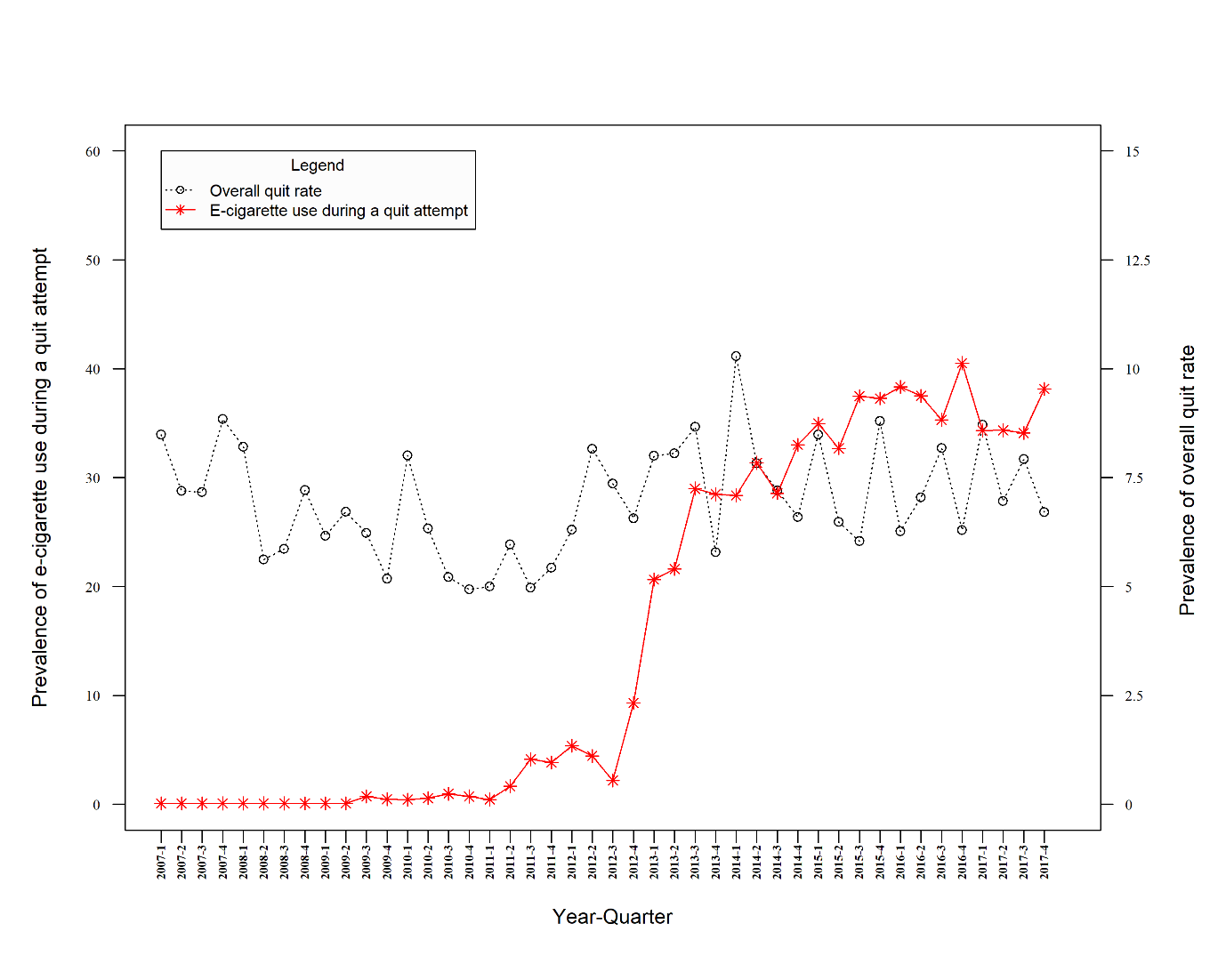


**Supplementary Figure 3:** Quarterly prevalence of overall quit rate and use of e-cigarettes during a quit attempt in England


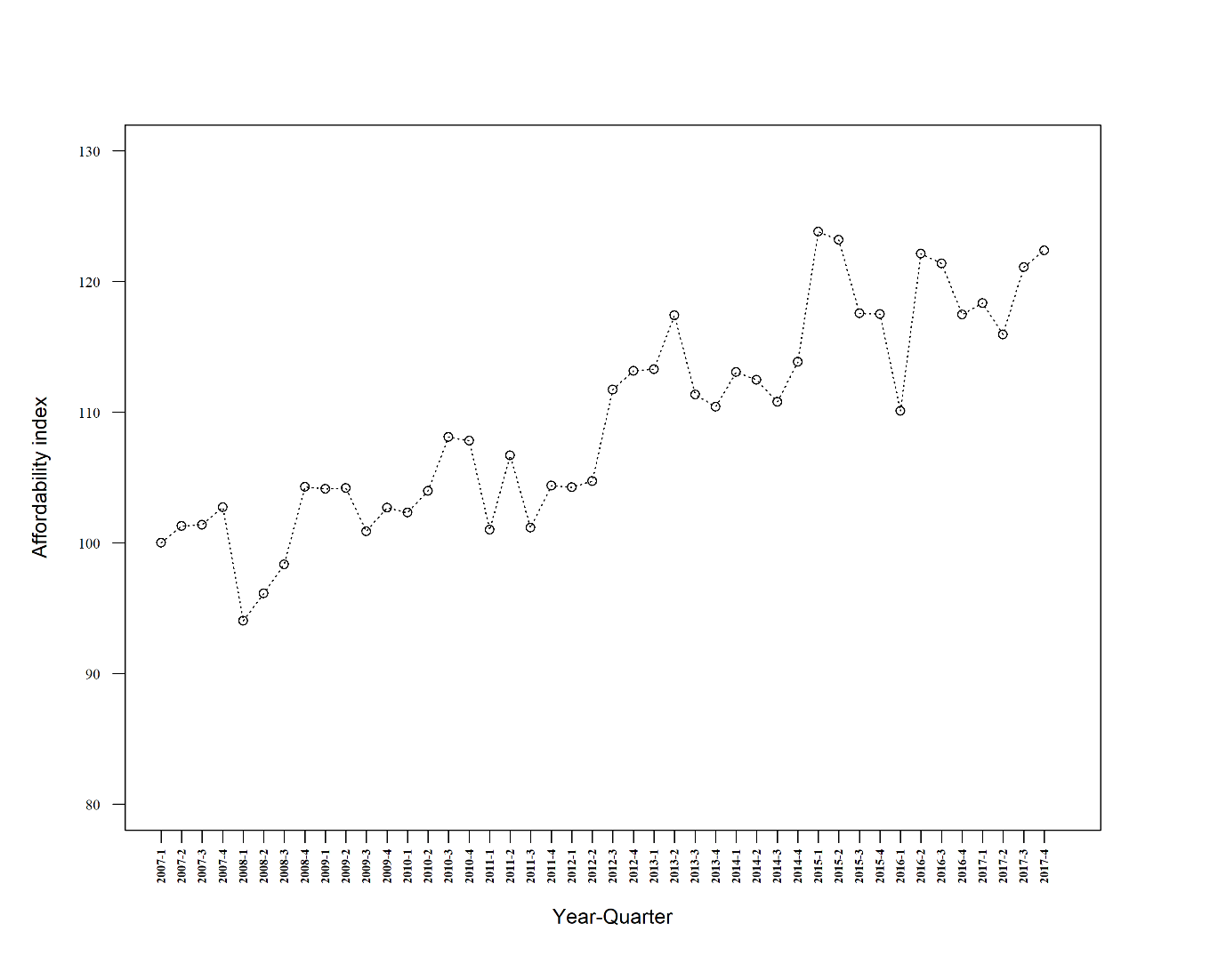


**Supplementary Figure 4:** Affordability index
